# *V*_TH_-Adjustable p-Channel GaN Field-Effect Transistor with an Inserted n-GaN Layer

**DOI:** 10.3390/mi17080913

**Published:** 2026-07-29

**Authors:** Yuheng Liu, Tao Zhang, Huake Su, Jiahao Chen, Xiangdong Li, Shengrui Xu, Zeyang Ren, Weidong Zhu, Yu Du, Yue Hao, Jincheng Zhang

**Affiliations:** 1 State Key Laboratory of Wide-Bandgap Semiconductor Devices and Integrated Technology, School of Microelectronics, Xidian University, Xi’an 710071, China; 2 Guangzhou Wide Bandgap Semiconductor Innovation Center, Guangzhou Institute of Technology, Xidian University, Guangzhou 510555, China

**Keywords:** GaN, p-channel field-effect transistor, threshold voltage, n-GaN layer, enhancement-mode operation

## Abstract

In this work, a novel Schottky-gated p-channel GaN field-effect transistor (PFET) with a tunable n-GaN sub-gate layer is investigated. Terminal-current analysis under the actual drain-bias condition shows that the gate-current contribution remains limited within the defined effective operating range of V_GS_ ≥ −3.2 V, whereas gate-related current becomes significant at more negative gate biases. Carrier-resolved and spatial current analyses further confirm that, within this operating range, the drain current is predominantly carried by holes through an interfacial hole channel near the p-GaN/AlGaN heterointerface. Benefiting from the intentionally introduced p–n junction beneath the groove gate, the built-in electric field effectively depletes the p-GaN channel, enabling a robust transition from depletion-mode to enhancement-mode (E-mode) operation. By precisely scaling the n-GaN layer thickness (0–5 nm) and donor concentration (3.0 × 10^17^ cm^−3^ to 3.0 × 10^19^ cm^−3^), the buried p-n junction modulates the depletion condition and hole distribution beneath the gate. The optimized device exhibits a significantly improved subthreshold swing (SS) of 348 mV/dec, while maintaining a stable I_ON_/I_OFF_ ratio on the order of 10^2^. This tunable sub-gate architecture provides a highly flexible platform for optimizing E-mode GaN PFETs, showing great promise for high-performance complementary logic applications.

## 1. Introduction

Due to the wide bandgap, high breakdown field, and superior thermal stability, gallium nitride (GaN) has been perceived as a promising candidate for next-generation electronic and optoelectronic devices. With the rapid development of high-power electronics and integrated circuits, there is an increasing demand for GaN-based devices that can support high-efficiency, high-switching-speed, and high-temperature operation [1,2,3,4,5,6,7,8]. In commercial applications such as power converters, radio-frequency systems and electric vehicle power electronics, GaN-based complementary circuits have demonstrated significant advantages [9,10,11]. Although comprehensive studies have been conducted on n-channel transistors, the development of reliable and high-performance p-channel devices remains challenging [12,13,14,15,16,17].

For all GaN complementary metal oxide semiconductor circuits, high-performance p-channel field-effect transistors (PFETs) are indispensable [12,18]. Various device structures have been proposed to improve hole transport and enhance device performance, including heterostructure engineering and multi-channel designs. In particular, Schottky-gated p-channel GaN field-effect transistors, where the gate voltage modulates the depletion region and effectively controls the hole conduction channel, have exhibited excellent electrical characteristics and enhancement-mode characteristics [19]. Most reported p-channel GaN transistors exhibit limited controllability. Achieving both depletion-mode and enhancement-mode operation within a single device structure and within the same device platform remains challenging. Flexible control of the threshold voltage is therefore essential for practical circuit applications [20,21,22,23,24]. Junction-gate depletion control has been demonstrated in GaN n-channel devices and may offer a promising route to improve threshold modulation and off-state leakage in p-channel GaN transistors [25].

In this work, a novel Schottky-gated p-channel GaN field-effect transistor structure is proposed and systematically investigated through device simulation. This device incorporates a tunable n-GaN layer beneath the gate region, which plays an important role in modulating the depletion region and hole distribution. By adjusting the thickness and doping concentration of the n-GaN layer, the controllable transition between depletion-mode and enhancement-mode operation is demonstrated, improving the tunability of p-channel devices while such tunability benefits the realization of GaN-based complementary circuits, where precise control of the threshold voltage is essential. The electrical characteristics of the proposed device are analyzed and presented in detail, including transfer characteristics, transconductance characteristics and output characteristics. Furthermore, the influence of the n-GaN layer thickness and doping concentration on the device performance is systematically studied. Additionally, the underlying physical mechanisms are further clarified through energy band diagrams and hole distribution analysis beneath the gate region.

## 2. Materials and Methods

**Figure 1:**
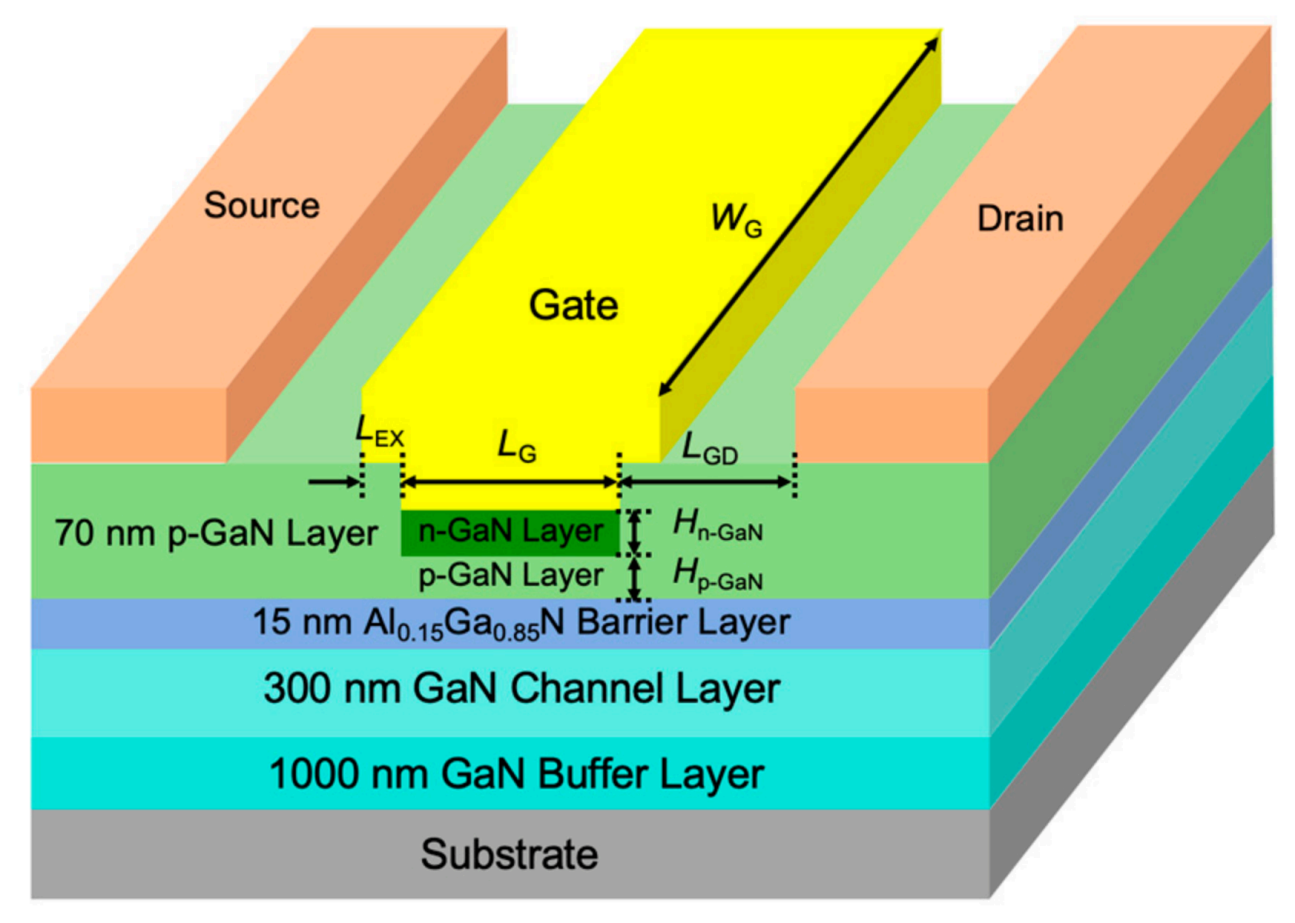
Cross-sectional view of the p-channel GaN FET with an inserted n-GaN layer.

The cross-sectional view of the schematic structure and dimensions of the proposed device are illustrated in Figure 1, consisting of a sapphire substrate, a 1 μm GaN buffer layer, a 300 nm undoped GaN channel layer for electrons, a 15 nm AlGaN barrier layer with an Al composition of 0.15, a 70 nm p-GaN layer with a hole concentration of $3.0\;\times \;10^{18}{\;\mathrm{cm}}^{-3}$, and an n-GaN layer with a thickness ranging from 0 nm to 5 nm and an electron concentration of $3.0\;\times {\;10}^{17}$ to $3.0\;\times \;10^{19}\;{\mathrm{cm}}^{-3}$ from bottom to top. A Schottky gate metal is deposited on top of the device, controlling the channel conduction. Meanwhile, the designed p-channel GaN field-effect transistor possess a gate foot length (L_G_) of 300 nm, which is located at the center of the active region, a gate extension length(L_EX_) of 600 nm, a depth of groove gate of 55 nm, and a gate-to-drain length (L_GD_) of 1.25 μm [26]. Unlike conventional Schottky-gated p-channel GaN transistors, an n-type GaN layer is intentionally introduced under the gate region, forming a p-n junction at the interface between the n-GaN and p-GaN layers.

The introduction of this n-GaN layer effectively modulates the depletion region under the gate. The built-in electric field generated by the p-n junction can significantly affect the hole distribution in the p-GaN channel, and as a result, the conduction characteristics of the device could be effectively controlled by the structural parameters of the n-type layer.

To fully and accurately investigate the electrical characteristics of the designed device, two-dimensional device simulations were carried out using a professional Technology Computer-Aided Design (TCAD) simulator. In order to accurately describe the physical behavior of the device, several physical models were incorporated in the simulation. These models include carrier drift-diffusion transport, Shockley–Read–Hall recombination, and Fermi–Dirac statistics. In addition, appropriate mobility models were adopted to account for the carrier transport in GaN-based materials. To account for gate leakage, a work-function-defined Schottky contact was used to describe thermionic-emission transport; the Universal Schottky Tunneling (UST) model was employed for tunneling through the Schottky barrier; and the TRAP.TUNNEL model, together with explicitly defined donor- and acceptor-like deep-level traps, was used to describe trap-assisted tunneling. Surface recombination at the Schottky gate contact was also included. Meanwhile, the source and drain contacts were defined as ohmic contacts, and tungsten (W), with an assumed work function of 4.6 eV, was used as the Schottky gate metal. To evaluate the impact of the n-GaN layer on device performance, systematic simulations were performed by varying its thickness and doping concentration. For each structure, transfer characteristics and output characteristics were extracted and compared.

Furthermore, by examining the energy band diagrams and hole concentration under the gate region, the electrical characteristics were analyzed. Those analyses provide insight into how the n-GaN layer parameters influence the depletion condition and the formation of the hole conduction channel.

## 3. Results and Discussion

**Figure 2:**
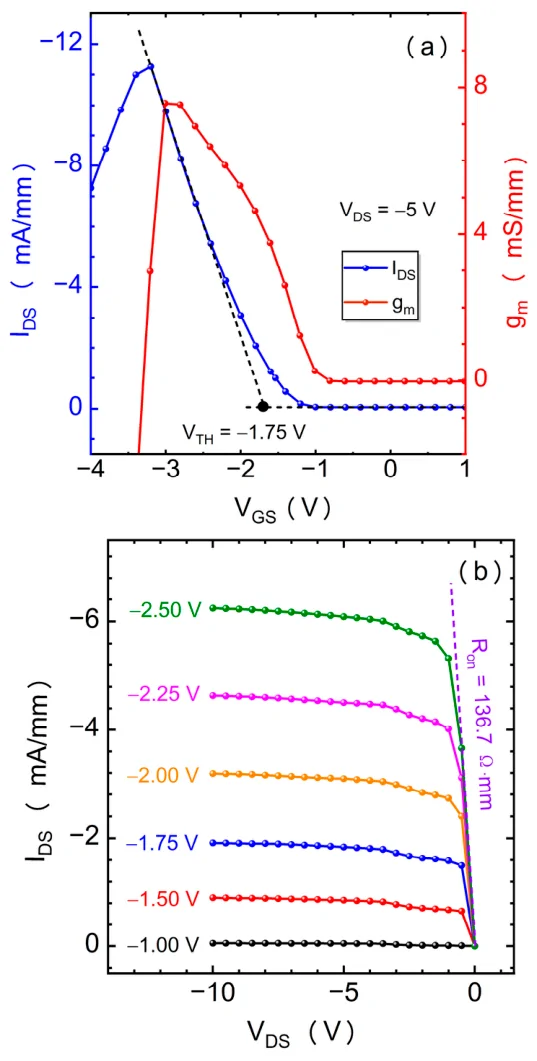
(**a**) Transfer characteristics and transconductance, and (**b**) output characteristics of GaN PFET with a groove gate with a 0 nm n-GaN layer and an electron concentration of $3.0\times 10^{17}\;{cm}^{-3}$.

To provide a baseline for comparison, a device with a 0 nm n-GaN inserted layer with an electron concentration of $3.0\times 10^{17}\;{cm}^{-3}$ was designed. Figure 2a shows the transfer characteristics and corresponding transconductance of this device at a drain voltage of −5 V. As the gate voltage decreases, the magnitude of the negative drain current gradually increases, indicating the formation and strengthening of the hole conduction channel. From the transfer curve, the threshold voltage of the device is extracted to be approximately −1.75 V. The output characteristics are shown in Figure 2b, with the gate voltage swept from −1 V to −2.5 V. As the magnitude of the negative gate voltage increases, the drain current increases correspondingly in the negative direction, demonstrating the typical output behavior of a p-channel FET. The curves exhibit a linear region at low drain voltage and gradually enter the saturation region as the magnitude of drain voltage increases. Based on the linear region, the on-state resistance was extracted. The calculated on-state resistance is approximately 136.7 Ω·mm, which provides a useful reference for evaluating the electrical performance of the proposed structure in the following sections.

**Figure 3:**
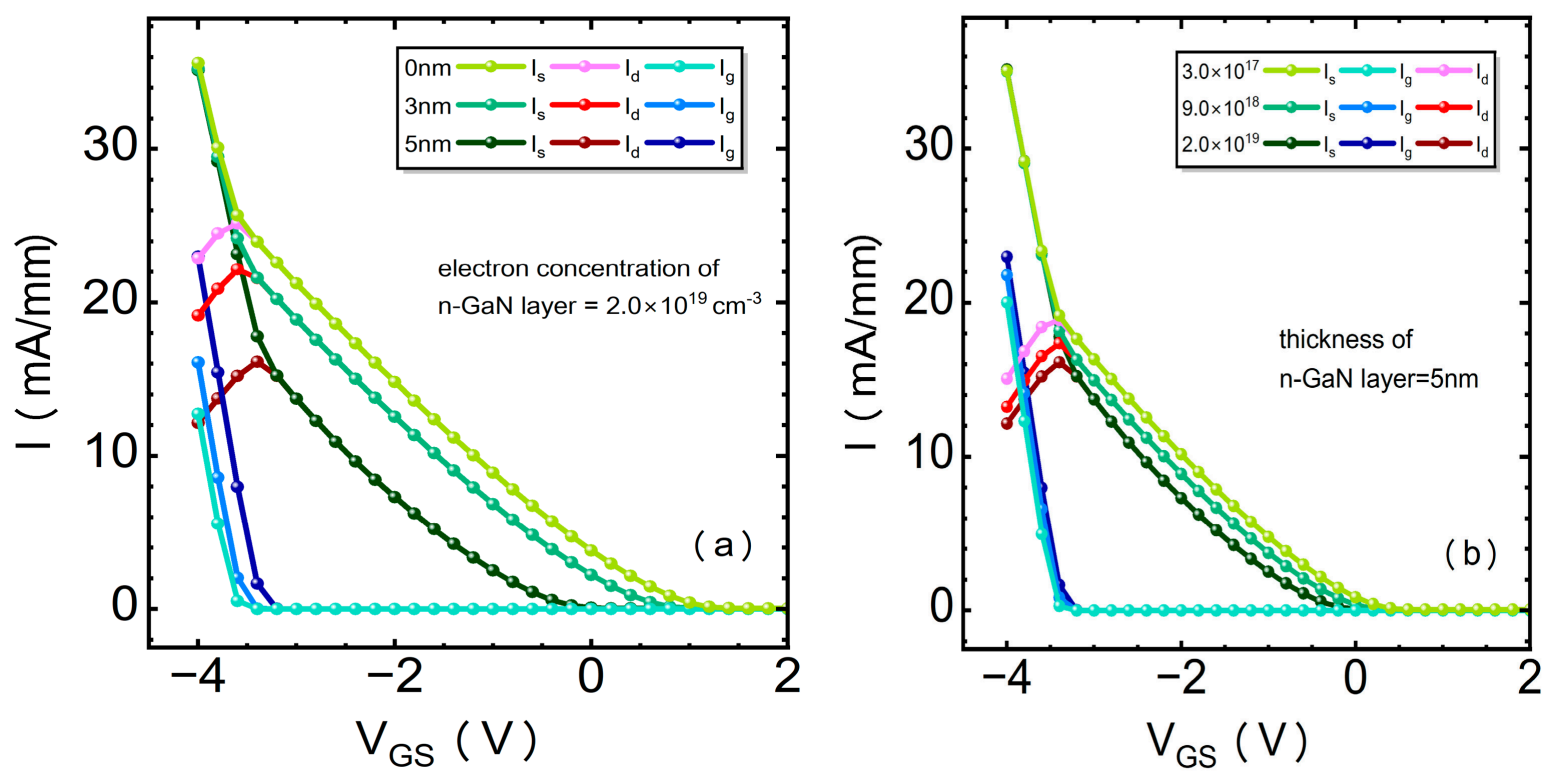
Terminal-current magnitudes as functions of $V_{GS}$ at $V_{DS}=-5\;V$ for representative devices with (**a**) n-GaN layer thicknesses of 0 nm, 3 nm, and 5 nm at a fixed donor concentration of $2.0\;\times \;10^{19}\;{\mathrm{cm}}^{-3}$, and (**b**) donor concentrations of $3.0\;\times {\;10}^{17}\;{\mathrm{cm}}^{-3}$, $9.0\;\times \;10^{18}\;{\mathrm{cm}}^{-3}$, and $2.0\;\times \;10^{19}\;{\mathrm{cm}}^{-3}$ at a fixed n-GaN layer thickness of 5 nm.

A slight degradation of $I_{d}$ is observed in the transfer characteristics in Figure 2a under strongly negative gate bias, which is attributed to the turn-on of the parasitic diode in the gate stack. Therefore, before evaluating the effects of the n-GaN layer thickness and donor concentration, the terminal-current characteristics were examined to determine the effective low-gate-current operating range of the device. Figure 3a,b show terminal-current magnitudes as functions of $V_{GS}$ at $V_{DS}=-5\;V$ for representative devices with different n-GaN layer thicknesses and electron concentrations. Using the signed terminal-current convention, the calculated currents satisfy $I_{d}+I_{g}+I_{s}\approx 0$ throughout the investigated bias range. Figure 3a shows that increasing the n-GaN layer thickness enhances the relative gate-current contribution under strongly negative gate bias. For the 5 nm devices in Figure 3b, decreasing the donor concentration reduces the gate-current contribution to some extent. These results illustrate that both the n-GaN layer thickness and donor concentration influence the gate-current blocking capability.

For devices containing an n-GaN interlayer, the lateral variation in the channel potential results in different bias conditions across the buried p-n junction, with the source-side junction driven toward forward bias and the drain-side junction remaining reverse-biased. At $V_{DS}=-5\;V$, $|I_{g}|$ remains much smaller than $|I_{d}|$ at a low-to-moderate negative gate bias, indicating that lateral channel transport dominates the drain current. To quantitatively evaluate the gate-current contribution, $|I_{g}/I_{d}|\leq 0.01$ was adopted as the criterion, yielding a conservative negative gate-voltage boundary of $V_{GS}=-3.2\;V$. Therefore, the effective operating range was defined as $V_{GS}\geq -3.2\;V$ within the investigated bias range. At more negative gate biases, $|I_{g}|$ increases rapidly and becomes comparable to $|I_{d}|$, leading to current redistribution among the three terminals. Consequently, the peak and subsequent decrease in the drain current should not be interpreted as a purely intrinsic channel response. The subsequent device-parameter extraction was therefore performed within the defined operating range.

**Figure 4:**
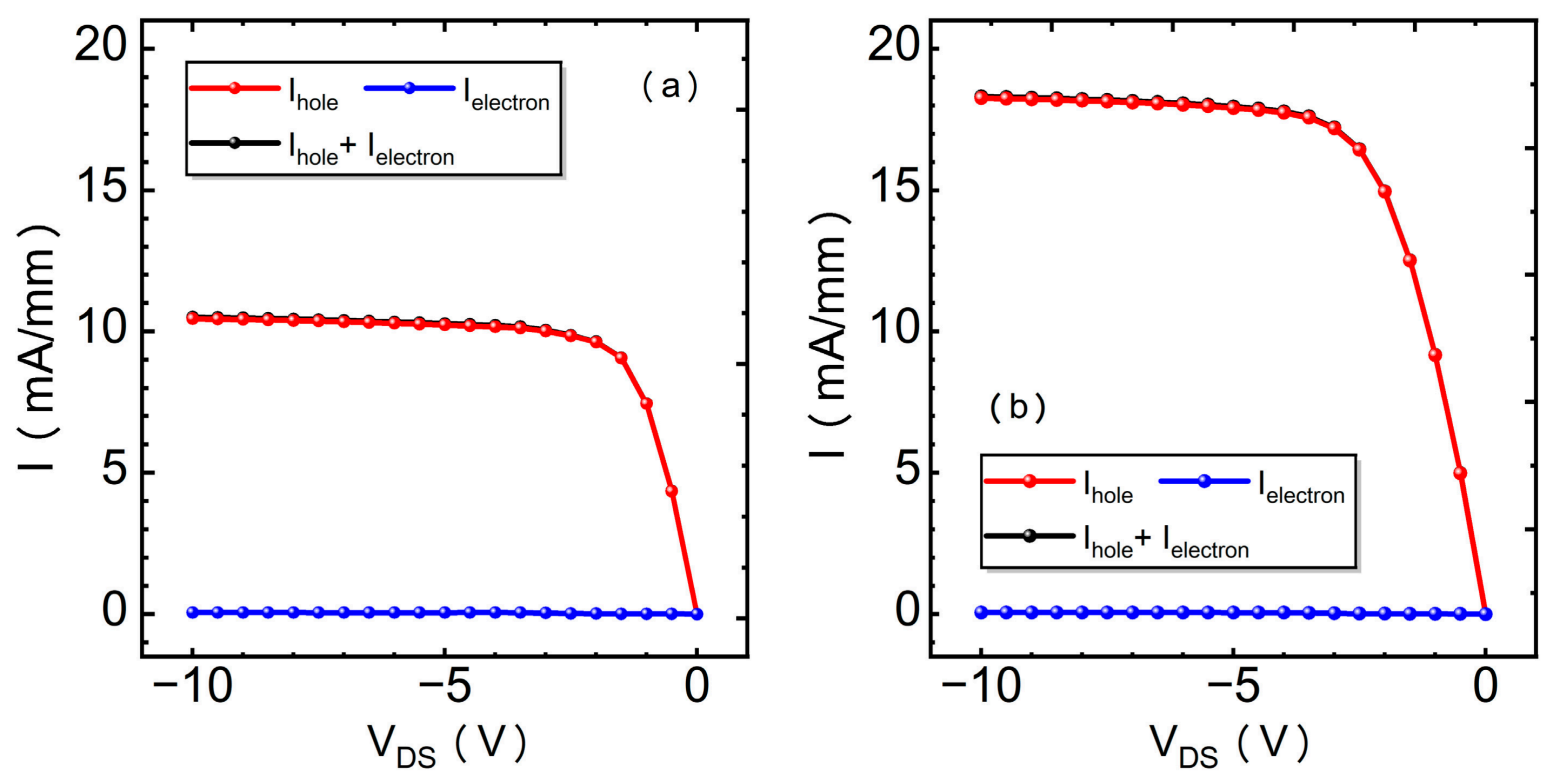
Carrier-resolved drain-current characteristics as functions of $V_{DS}$ at $V_{GS}=-2.5\;V$ for devices with n-GaN layer thicknesses of (**a**) 5 nm and (**b**) 0 nm.

To further identify the carrier type and conduction path responsible for the drain current within the defined effective operating range, the electron- and hole-current components were separately extracted. Figure 4a,b show the carrier-resolved drain currents as functions of $V_{DS}\;$ at $V_{GS}=-2.5\;V$ for devices with n-GaN layer thicknesses of 5 and 0 nm, respectively. For both devices, the hole-current component almost completely overlaps the total drain current over the investigated drain-voltage range, whereas the electron-current component remains negligible. These results indicate that, under the selected low-gate-current operating condition, the drain current is predominantly carried by holes rather than being dominated by electron injection.

**Figure 5:**
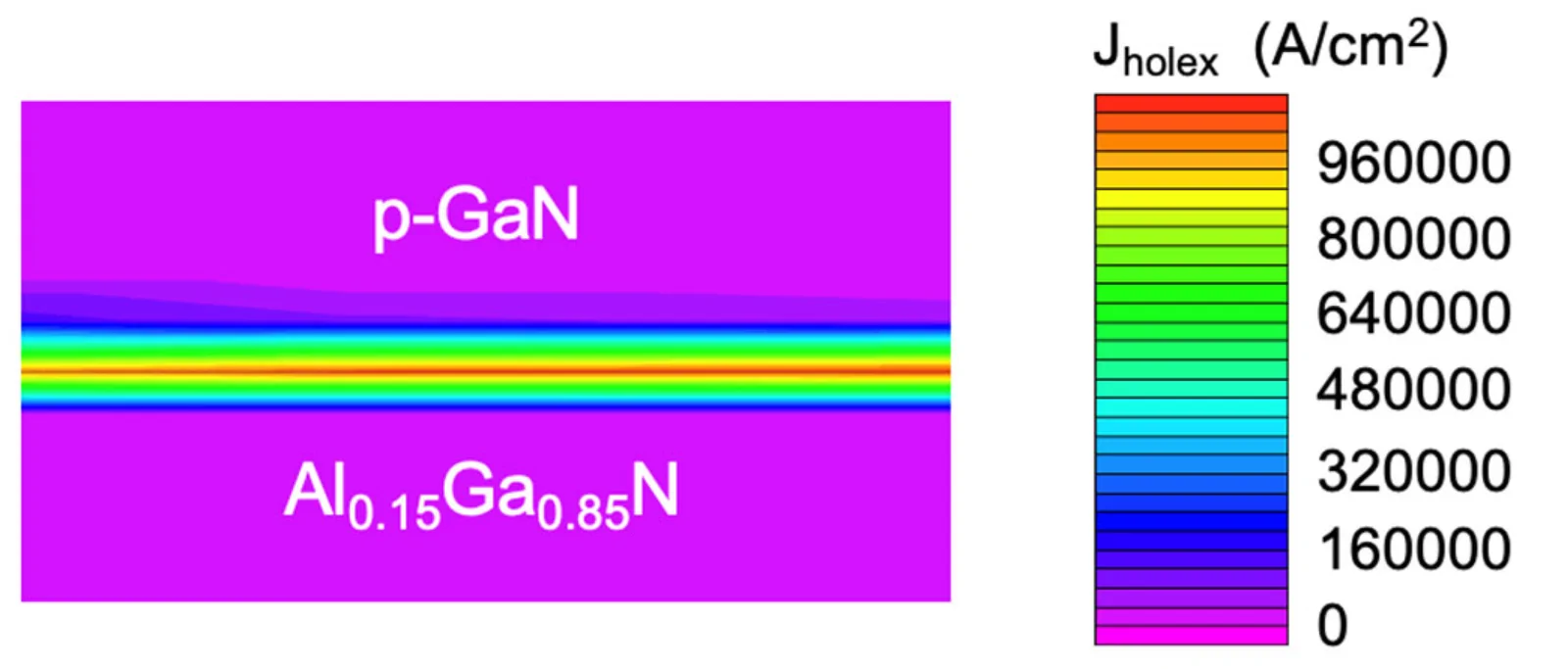
Spatial distribution of the lateral hole-current density with a 5 nm inserted n-GaN layer at $V_{DS}=-5\;V$.

The spatial distribution of the lateral hole-current density was further examined for the device with a 5 nm n-GaN interlayer at $V_{GS}=-2.5\;V$ and $V_{DS}=-5\;V$, as shown in Figure 5. The lateral hole-current density is concentrated within a narrow region near the p-GaN/AlGaN heterointerface, confirming that the intended interfacial hole channel constitutes the primary lateral conduction path. Therefore, within the defined effective operating range, the drain current can be mainly attributed to lateral hole transport through the interfacial channel rather than electron transport or uniform bulk conduction through the entire p-GaN layer.

**Figure 6:**
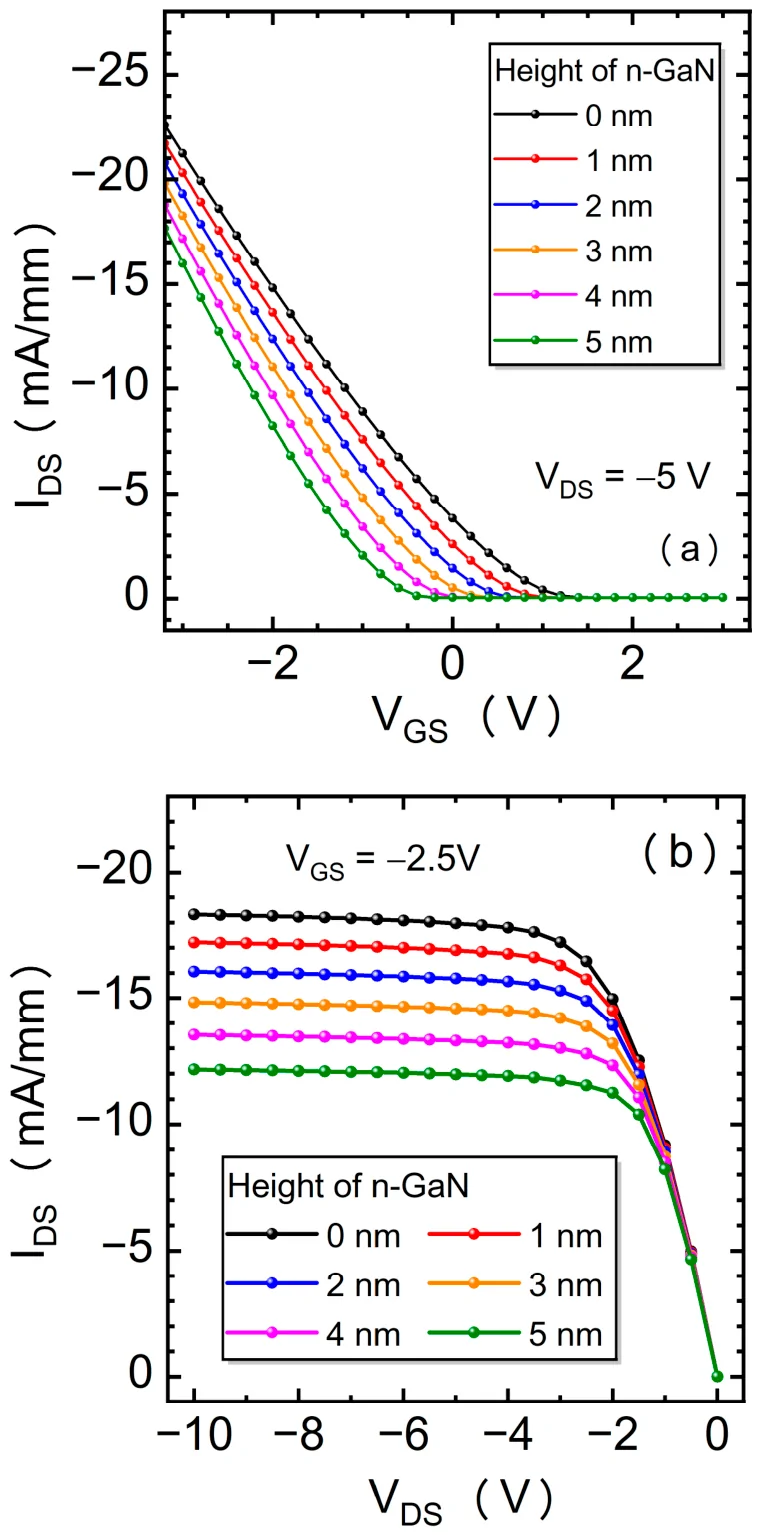

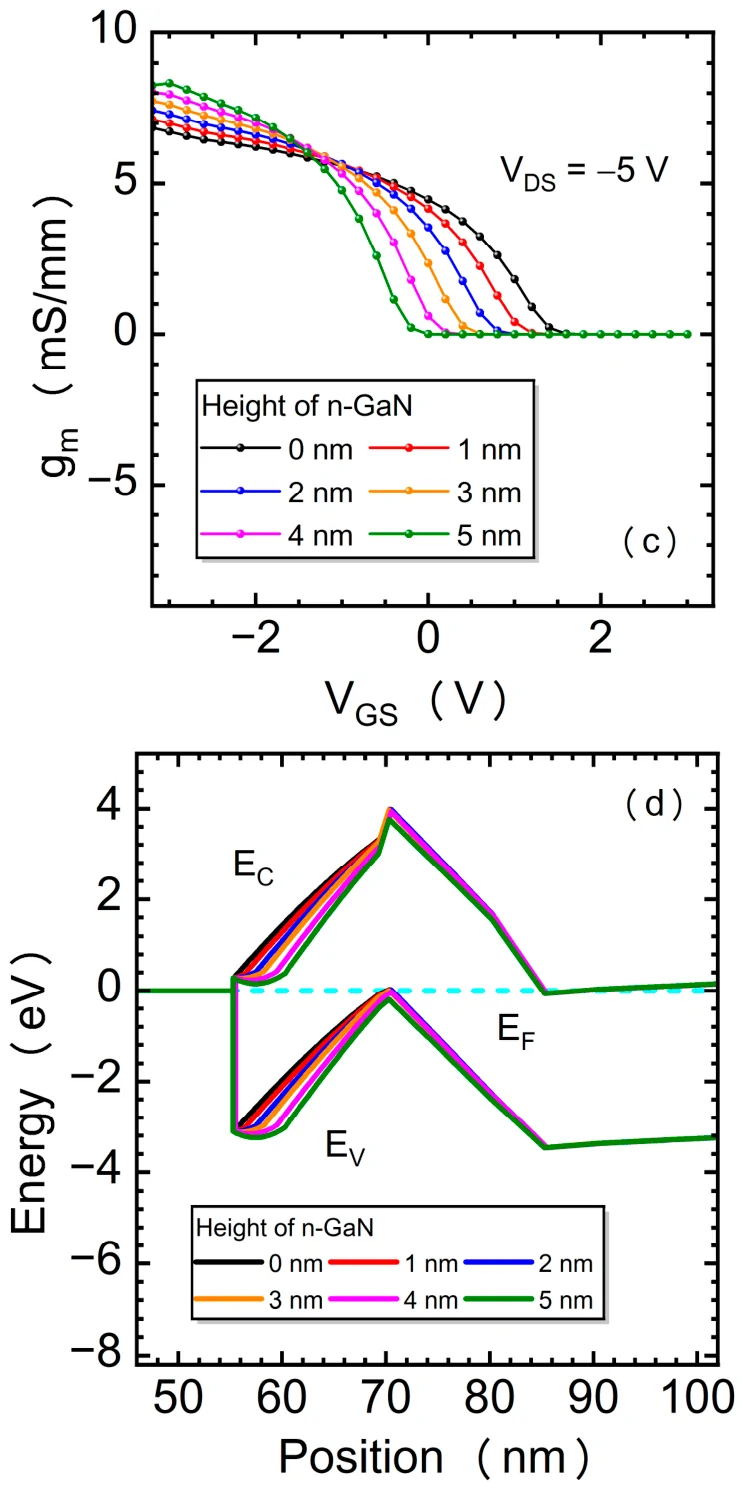
(**a**) Transfer characteristics, (**b**) output characteristics and (**c**) transconductance characteristics of the studied GaN FET with various n-layer thicknesses. (**d**) Energy band diagrams under the gate region with various n-layer thicknesses.

To further investigate the effectiveness of the structure, the thickness of the n-GaN layer beneath the gate metal was varied from 0 nm to 5 nm while keeping other parameters unchanged. Figure 6a shows the transfer characteristics for different n-GaN layer thicknesses. It can be observed that as the thickness increases from 0 nm to 5 nm, the device operation gradually shifts from depletion mode to enhancement mode. For a thickness of 0 nm, the device exhibits clear depletion-mode behavior, because the channel is already conductive at zero gate bias. As the thickness increases, the threshold voltage shifts toward more negative values, and the channel becomes fully depleted at zero gate voltage, resulting in enhancement-mode operation. Figure 6b presents the output characteristics with different n-layer thicknesses. The absolute value of the drain current decreases as the thickness increases, which can be attributed to the relatively strong depletion effect and the low hole concentration in the channel. The corresponding transconductance curves are shown in Figure 6c. Those results indicate that by properly tuning the n-layer thickness, a flexible transition between the depletion mode and enhancement mode can be achieved, providing an effective approach for device design optimization.

To quantitatively evaluate the switching performance, the on/off current ratio (I_ON_/I_OFF_) and subthreshold swing (SS) were extracted from the transfer characteristics for varied n-layer thickness. The drain current at $V_{GS}=-3.2\;V$ was taken as I_ON_ and the drain current at $V_{GS}=3\;V$ was taken as $I_{OFF}$. The results show that the I_ON_/I_OFF_ ratio remains on the order of $10^{2}$ for all structures, and specifically, the I_ON_/I_OFF_ ratio decreases from approximately $4.5\;\times \;10^{2}$ at 0 nm to about $3.7\;\times \;10^{2}$ at 5 nm for the enhanced depletion effect induced by the thicker n-layer. It is observed that the SS value shows a significant change with increasing n-layer thickness, decreasing from about 1400 mV/dec at 0 nm to about 600 mV/dec at 5 nm, demonstrating a stronger control over the channel as the n-layer thickness increases, providing a significant advantage for device optimization and potential applications in reconfigurable and complementary circuits [27]. The energy band diagrams under the gate are further analyzed for various n-layer thicknesses in Figure 6d. For the structure without the n-layer (0 nm), the bands exhibit relatively weak bending, indicating limited depletion in the p-GaN region and a higher hole concentration near the channel. As the thickness increases, a pronounced band bending appears due to the formation of a p–n junction. This results in an expanded depletion region and a reduced carrier concentration under the gate. The increased band modulation with thicker n-layers confirms the strengthened built-in electric field and enhanced gate control capability.

To visually validate the underlying physical mechanisms deduced from the electrical characteristics, the 2D hole concentration contour plots beneath the gate region were extracted.

**Figure 7:**
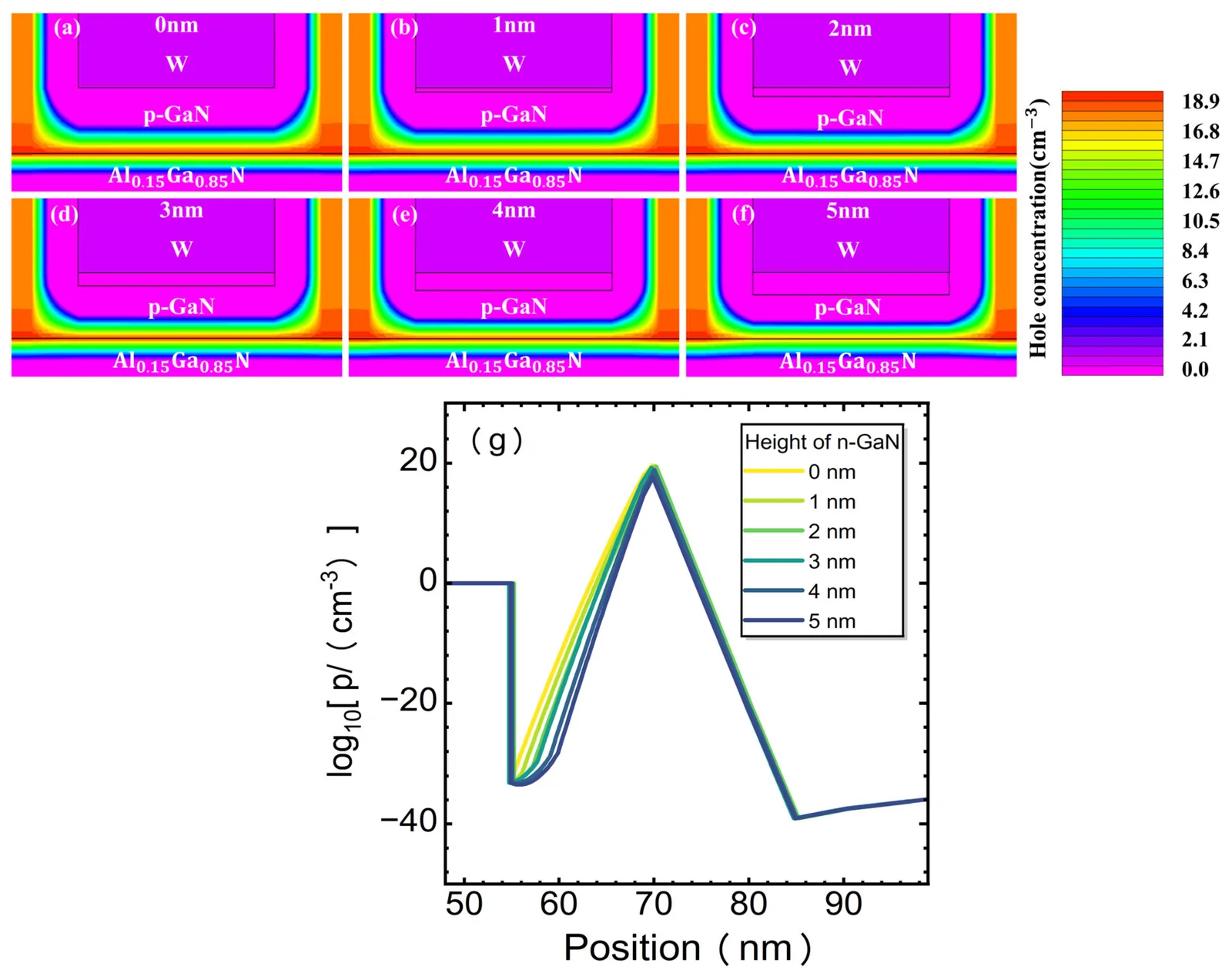
Distribution of hole concentration under the groove gate with various n-layer thickness of (**a**) 0 nm, (**b**) 1 nm, (**c**) 2 nm, (**d**) 3 nm, I 4 nm, and (**f**) 5 nm. (**g**) Cutline profiles.

Figure 7a–f illustrate the evolution of the hole distribution as the n-GaN layer thickness increases from 0 nm to 5 nm and show that the main conduction path in the proposed device is the two-dimensional hole gas (2DHG), formed by the accumulated holes near the p-GaN/AlGaN heterointerface, rather than bulk conduction through the entire p-GaN layer. Therefore, the hole concentration distributions mainly reflect the modulation of the interfacial hole channel by the inserted n-GaN layer. In the structure without the n-layer (0 nm), the depletion region is relatively shallow, leaving a highly concentrated conductive hole channel in the p-GaN layer. However, as the n-GaN layer becomes thicker, the physical volume of the n-type region expands. The depletion region extends significantly deeper into the adjacent p-GaN layer. This structural modulation effectively pushes the high-concentration hole profile away from the gate and thoroughly depletes the channel, facilitating the transition to enhancement-mode (E-mode) operation. The corresponding cutline profiles in Figure 7g further support the conclusion that the main conduction channel is the 2DHG instead of the other conduction mechanisms.

To further investigate the tunability of the proposed structure, the impact of the n-GaN layer doping concentration on the device performance was evaluated. The electron concentration was varied from $3.0\;\times \;10^{17}\;{\mathrm{cm}}^{-3}$ to $3.0\;\times {\;10}^{19}\;{\mathrm{cm}}^{-3}$, while all geometric dimensions were kept unchanged.

**Figure 8:**
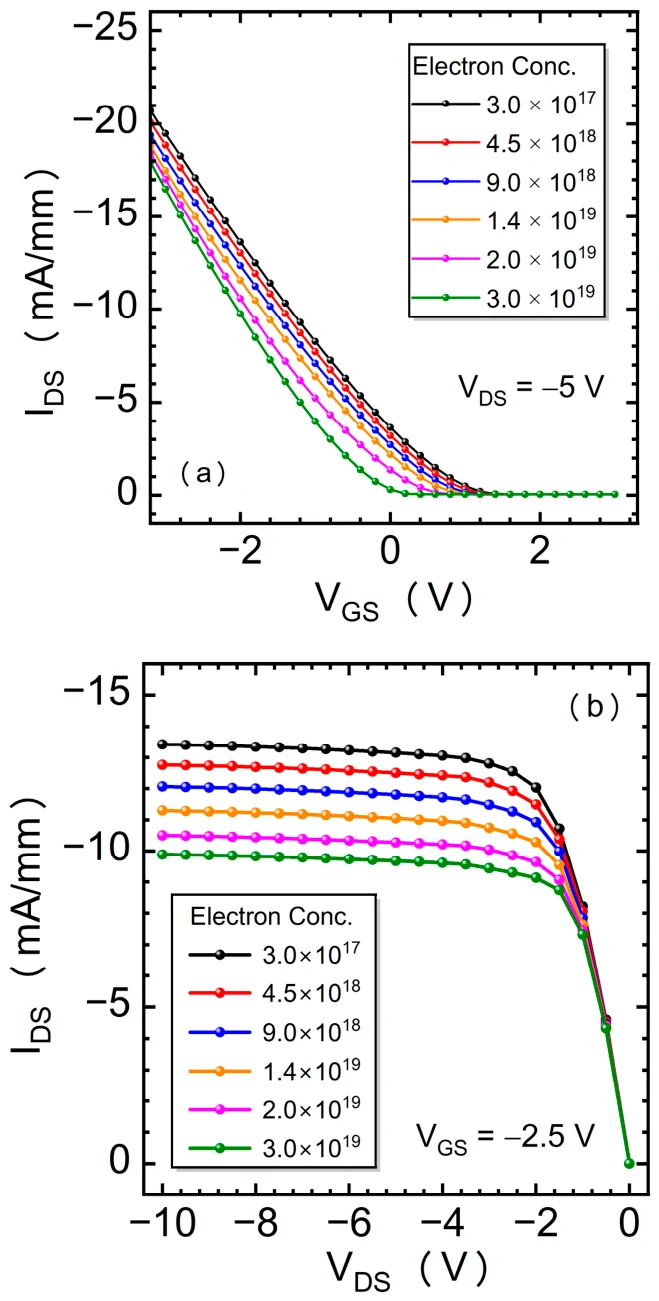

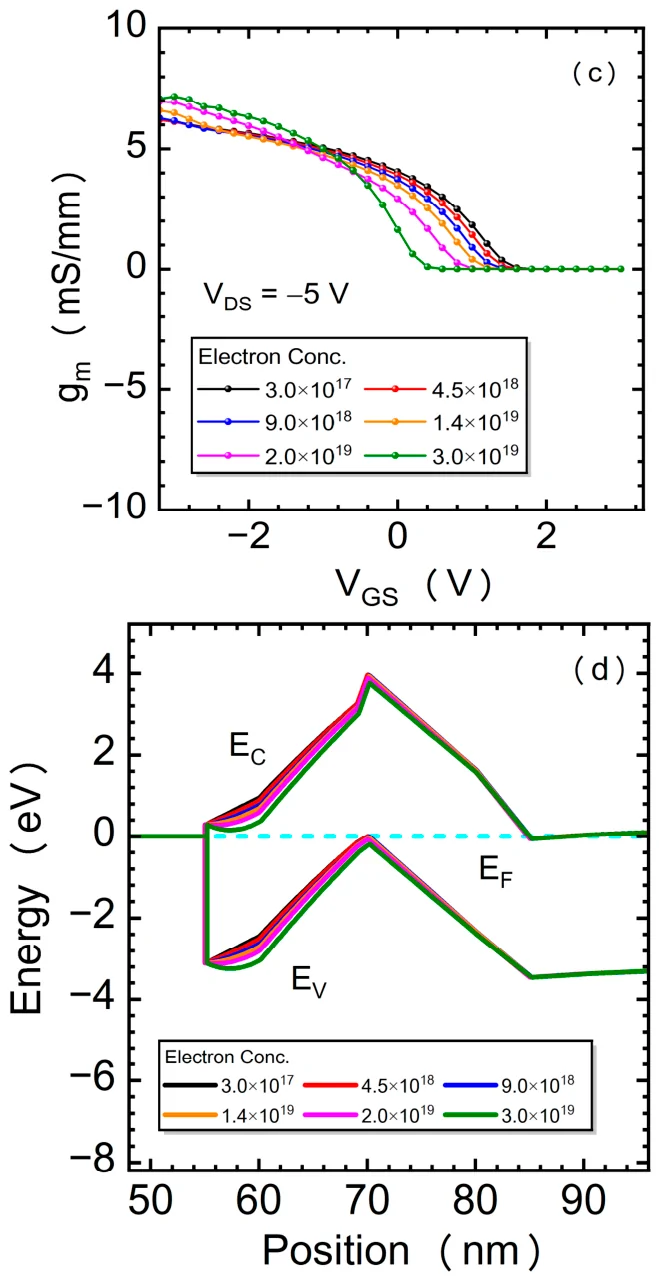
(**a**) Transfer characteristics, (**b**) output characteristics and (**c**) transconductance characteristics of the studied GaN FET with various electron concentrations. (**d**) Energy band diagrams under the gate region with different electron concentrations.

Figure 8a shows the transfer characteristics of the proposed structure at a drain-to-source voltage ($V_{\mathrm{DS}}$) of −5 V across various electron concentrations. As it increases, the threshold voltage ($V_{\mathrm{TH}}$) exhibits a continuous negative shift, which indicates that the depletion effect is enhanced by the increasing electron concentration, and thereby realizing a stronger enhancement-mode (E-mode) operation. Figure 8b illustrates the output characteristics at a fixed gate-to-source voltage ($V_{\mathrm{GS}}$) of −2.5 V. With an increase in electron concentration, the absolute magnitude of the saturation drain current ($I_{\mathrm{DS}}$) decreases, which is attributed to the strengthened built-in electric field induced by the heavily doped n-GaN layer. This electric field depletes a larger fraction of holes in the p-GaN channel. The transconductance characteristics, shown in Figure 8c, exhibit a negative voltage shift consistent with the transfer curves. The device maintains a stable peak transconductance across the evaluated doping range, indicating reliable switching performance.

To quantitatively evaluate the switching characteristics under varying doping levels, the on/off current ratio and the subthreshold swing were similarly extracted from the transfer curves as well. Specifically, the ratio exhibits a slight reduction from approximately $3.85\;\times \;10^{2}$ at a concentration of $3.0\;\times {\;10}^{17}\;{\mathrm{cm}}^{-3}$ to $3.84\;\times \;10^{2}$ at $\;3.0\;\times \;10^{19}\;{\mathrm{cm}}^{-3}$, reflecting the intrinsically enhanced depletion mechanism beneath the gate. Meanwhile, the subthreshold swing varies between 348 and 388 mV/dec. These parameters indicate that modulating the donor concentration effectively strengthens gate electrostatic control and tunes the threshold voltage without compromising fundamental switching performance. To analyze the underlying physical mechanisms, the energy band diagrams beneath the gate region for varying electron concentrations are extracted, as plotted in Figure 8d. As the electron concentration increases, the conduction band and the valence band both exhibit stronger bending. Consequently, the expanded depletion width induced by the higher electron concentration provides improved electrostatic control over the channel.

**Figure 9:**
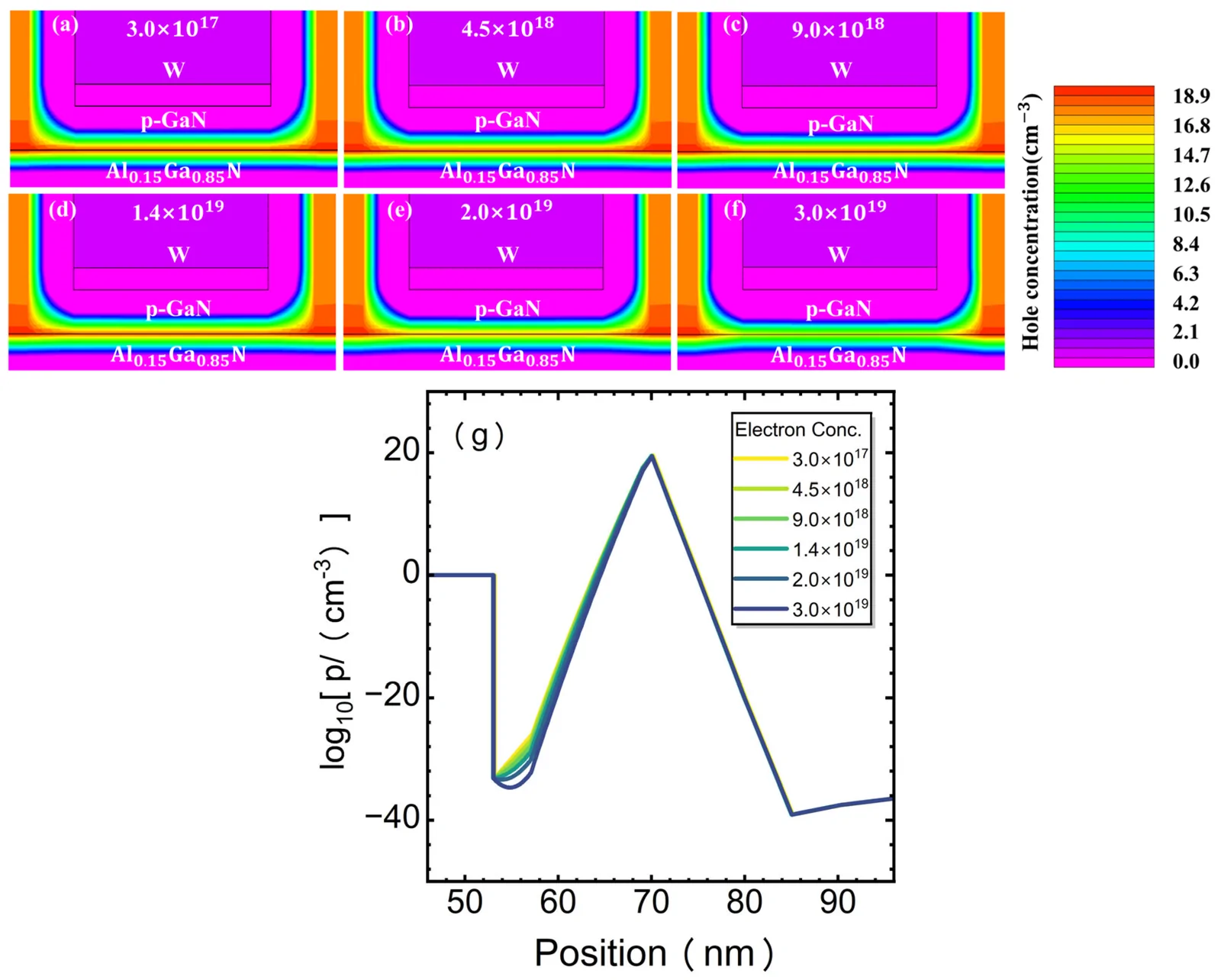
Distribution of hole concentration under the groove gate with various electron concentrations of (**a**) $3.0\times 10^{17}\;{cm}^{-3}$, (**b**) $4.5\times 10^{18}\;{cm}^{-3}$, (**c**) $9.0\times 10^{18}\;{cm}^{-3}$, (**d**) $1.4\times 10^{19}\;{cm}^{-3}$, (**e**) $2.0\times 10^{19}\;{cm}^{-3}$, (**f**) $3.0\times 10^{19}\;{cm}^{-3}$. (**g**) Cutline profiles.

Similarly, Figure 9a–f illustrate the impact of the n-layer doping concentration on the microscopic carrier distribution. As the donor concentration in the n-GaN layer increases from $3.0\;\times \;10^{17}{\;\mathrm{cm}}^{-3}$ to $\;3.0\;\times \;10^{19}{\;\mathrm{cm}}^{-3}$, the built-in electric field at the p-n junction interface is substantially strengthened. Because the depletion width extends predominantly into the relatively lighter-doped side, this higher donor concentration aggressively widens the depletion region within the p-GaN channel. Consequently, the conductive hole channel is effectively pinched off, as visually evidenced by the progressive expansion of the fully depleted region. These microscopic carrier profiles physically corroborate the enhanced depletion effect, fundamentally explaining the negative threshold voltage shift and the robust gate control capability.

The corresponding cutline profiles in Figure 9g further provide a more quantitative view of this carrier redistribution. The extracted profiles show that all devices exhibit a similar overall hole distribution trend, indicating that the basic channel formation mechanism is maintained. Meanwhile, pronounced local variations can be observed near the inserted n-GaN layer and the p-GaN/AlGaN interface. As the donor concentration increases, the hole concentration in the depletion-affected region is further reduced, confirming that the enhanced n-type doping strengthens the local depletion effect in the p-channel region. These cutline profiles directly demonstrate that the inserted n-GaN layer modifies the spatial distribution of holes beneath the gate.

## 4. Conclusions

In summary, a novel Schottky-gated p-channel GaN field-effect transistor featuring a tunable n-GaN sub-gate layer is proposed and systematically investigated. Terminal-current analysis at $V_{DS}=-5\;V$ shows that the gate-current contribution remains limited within the defined effective operating range of $V_{GS}\geq -3.2\;V$. By precisely engineering the n-layer thickness (up to 5 nm) and electron concentration (up to $3.0\;\times \;10^{19}\;{\mathrm{cm}}^{-3}$), the strengthened built-in electric field effectively pinches off the hole conduction at zero gate bias, enabling a controllable transition from depletion-mode to enhancement-mode operation. The optimized device exhibits a minimum subthreshold swing of approximately 348 mV/dec and the re-extracted on/off current ratio remains on the order of $10^{2}$. These results indicate that the optimized n-GaN doping level improves gate electrostatic control, although gate leakage under large negative bias remains an issue requiring further optimization. While grounded in rigorous physical simulations, these promising results establish a solid foundation for future experimental fabrication, offering a highly flexible methodology for developing advanced high-performance GaN-based complementary logic circuits.

## Data Availability

The original contributions presented in this study are included in the article. Further inquiries can be directed to the corresponding authors.
